# Weathered Coal-Immobilized Microbial Materials as a Highly Efficient Adsorbent for the Removal of Lead

**DOI:** 10.3390/molecules29030660

**Published:** 2024-01-31

**Authors:** Zile Jiao, Chunhua Gao, Jianhua Li, Jinjing Lu, Juan Wang, Lin Li, Xiaojing Chen

**Affiliations:** 1 College of Resources and Environment, Shanxi Agricultural University, Taiyuan 030031, China; jzl2573657313@163.com (Z.J.); jianhua0119@163.com (J.L.); nihaolujinjing@163.com (J.L.); wj2634430472@163.com (J.W.); lilin8129@163.com (L.L.); 2 Shanxi Province Key Laboratory of Soil Environment and Nutrient Resources, Taiyuan 030031, China; 3 Institute of Loess Plateau, Shanxi University, Taiyuan 030006, China

**Keywords:** weathered coal, immobilized microbial materials, lead, adsorption conditions, adsorption mechanism

## Abstract

Most research on immobilized microorganisms employs biomass charcoal as a carrier, but limited studies explore coal-based resources for microbial immobilization. Herein, lead-resistant functional strains were immobilized using weathered coal as a carrier, resulting in the development of a weathered coal-immobilized microbial material (JK-BW) exhibiting high efficiency in lead removal from solutions. A quadratic polynomial model for the adsorption capacity and adsorption rate of JK-BW on Pb^2+^ was developed using the Box-Behnken method to determine the optimal adsorption conditions. The Pb^2+^ adsorption mechanism of JK-BW was studied through batch adsorption and desorption experiments along with SEM-EDS, BET, FT-IR, and XPS analyses. Findings indicated that optimal conditions were identified at 306 K temperature, 0.36 g/L adsorbent dosage, and 300 mg/L initial solution concentration, achieving a peak adsorption performance of 338.9 mg/g (308 K) for the immobilized material, surpassing free cell adsorption by 3.8 times. Even after four cycles of repeated use, the material maintained its high adsorption capacity. Pb^2+^ adsorption by JK-BW involved monolayer chemisorption with ion exchange, complexation, precipitation, physical adsorption, and microbial intracellular phagocytosis. Ion exchange accounted for 22–42% and complexation accounted for 39–57% of the total adsorption mechanisms, notably involving exchanges with K, Ca, Na, and Mg ions as well as complexation with –OH, –COOH, CO–OH, –COOH, CO–, NH_2_, and the β-ring of pyridine for Pb^2+^ adsorption.

## 1. Introduction

With the development of various industries, including electroplating, printing, smelting, and ceramics, a large amount of industrial wastewater is directly discharged, resulting in excessive levels of heavy metals, such as copper, mercury, chromium, and lead, in water bodies [1]. Among these heavy metals, lead is classified as the first group of toxic-polluting elements and is characterized by strong bioaccumulation and persistent toxicity [2,3,4]. Lead can enter and be enriched in a human body through the food chain [5,6]. When a certain amount of lead is present in the body, the blood and nervous system can be severely damaged; for instance, lead consumption can have irreversible effects on the intellectual development of children [1,7]. Therefore, the problem of lead pollution urgently needs to be addressed.

Adsorption has become the most frequently used and widely applicable method for heavy metal wastewater treatment due to its advantages of low price, easy operation, and lack of secondary pollution [1,8,9,10]. Biosorbents that are represented by functional strains contain many active functional groups, which have great potential to combine with heavy metals. Biosorbents have become a focus of recent research [11]. To enhance the effect of microbial adsorption, many scholars have used microbial immobilization technologies to prepare immobilized microbial adsorption materials through physically or chemically fixing microorganisms on carriers [12,13]. Immobilized microorganisms show higher biological activity and adsorption capacity characteristics than free cells and are easily recycled and reused [14,15]. An et al. [16] used rice biochar to immobilize Pseudomonas L1 to remove Ni^2+^ from wastewater. The adsorption rate was 82.68%, which was 145% higher than that of free bacteria. Zhang et al. [17] used bamboo biochar to immobilize Pseudomonas to remove nitrite, and the adsorption rate still exceeded 95% after 10 times of repeated use. Huang et al. [18] used biochar to immobilize Bacillus cereus, and the results indicated that the adsorption effect of immobilized microorganisms on heavy metals is significantly higher than that of single microorganisms or biochar; this material easily realized solid–liquid separation in practical applications. However, most of these studies have focused on biomass carbon as the immobilization carrier, and there are few reports on coal-based resources as the immobilization carriers of microorganisms. Weathered coal (JK) is characterized by a reasonable pore size distribution, high humic acid content and abundant active functional groups, and it is nontoxic with good biocompatibility. The preparation of immobilized carriers from weathered coal can endow it with new potential for application.

Robalds et al. [19] classified the adsorption mechanism of immobilized microbial materials into two parts: active and passive adsorption. Active microorganisms can actively adsorb heavy metal ions to the cell surface or phagocytose and accumulate them in the body. Specifically, the adsorption mechanisms of immobilized microbial materials include the following (active and passive): intracellular accumulation, electrostatic and van der Waals forces [20], ion exchange with positively charged ions [21], coordination complexation with functional groups [22], precipitation [23], etc. But the relative contribution of these mechanisms remains unclear. In this study, an immobilized microbial material (JK-BW) exhibiting high efficiency for lead adsorption was prepared with weathered coal used as the carrier. The adsorption conditions were optimized with response surface methodology, and the adsorption mechanism was qualitatively and quantitatively analyzed with adsorption and desorption experiments and various characterization techniques. This study is expected to provide a material basis and theoretical support for microbial remediation of Pb^2+^ pollution.

## 2. Results and Discussion

### 2.1. Structural Characterization

**Figure 1:**
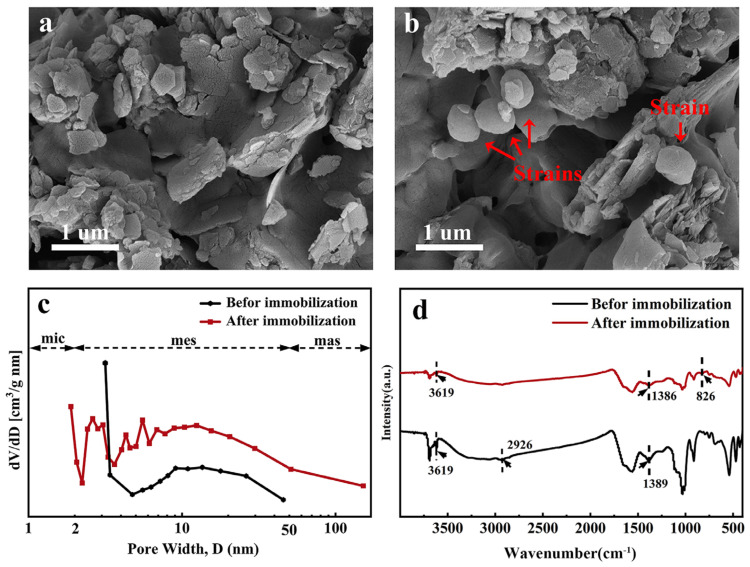
SEM images (**a**,**b**), pore size distributions (**c**) and FT-IR spectra (**d**) before determined and after immobilization.

SEM images before and after immobilization are shown in Figure 1a,b. Before immobilization (JK-B), SEM shows a relatively broken and compact sheet structure. After immobilization (JK-BW), it is obvious that the strain is loaded in the pores of the carrier in a cluster shape.

A pore size distribution diagram is shown in Figure 1c. The micropore area, mesopore area and pore volume of JK-B are 1.890 m^2^/g, 1.232 m^2^/g and 0.0040 cm^3^/g, respectively; those of JK-BW are 2.488 m^2^/g, 0.4540 m^2^/g and 0.0013 cm^3^/g, respectively (Table S2). Compared with JK-B, the mesopore area and pore volume of JK-BW decrease, while the micropore area increases, which is possibly because the bacteria are attached to fill the pores of JK-B. Zhang et al. [24] show that the porosity of the material is smaller because of cell occupation, and the structure is more complex, which is similar to the results in this paper.

FT-IR spectra before and after immobilization are presented in Figure 1d. Before immobilization, a broad peak at 3619 cm^−1^ appears corresponding to the O–H stretching vibration of the hydroxyl group [25]. In addition, some small narrow and sharp peaks can be clearly observed: at 2926 cm^−1^, the stretching for aliphatic C–H; the –COO^−^ or –OH peaks in phenolics were observed at around 1389 cm^−1^ [26]. After immobilization, the hydroxyl –OH vibrational peak was enhanced and –COO^−^ or –OH went from 1389 to 1386 cm^−1^, indicating that –OH and –COO^−^ were involved in the immobilization process. Also, the disappearance of aliphatic C–H and the appearance of a new absorption peak (corresponds to r–CH of furan or β-rings) at 826 cm^−1^ indicate the successful immobilization of the strain on the carrier and the involvement of chemistry.

### 2.2. Optimization of the Adsorption Conditions

BBD was used to optimize the adsorption conditions of JK-BW. The effects of the adsorption temperature, adsorption time, pH, adsorbent dosage and initial concentration of solution on the Pb^2+^ adsorption capacity and adsorption rate with JK-BW were investigated. The BBD parameters and results are shown in Table S1. The second-order polynomial equations were obtained by regression analyses as Equations (1) and (2), where *Y*_1_ and *Y*_2_ are the unit amount of Pb^2+^ adsorption (mg/g) and the removal rate (%) for JK-BW, and *X*_1_, *X*_2_, *X*_3_, *X*_4_ and *X*_5_ are the actual adsorption temperature, adsorption time, pH, adsorbent dosage and initial concentration of solution, respectively.

(1) $$\begin{array}{ll} Y_{1}= & -15052.71985+88.86X_{1}+24.12X_{2}+773.40X_{3}+1440.29X_{4}+0.46X_{5}\\ & +0.032X_{1}X_{2}-1.31X_{1}X_{3}-2.75X_{1}X_{4}+0.0060X_{1}X_{5}+9.78X_{3}X_{4}\\ & -0.15X_{3}X_{5}+1.43X_{4}X_{5}-0.14X_{1}^{2}-2.08X_{2}^{2}-44.31X_{3}^{2}-1747.37_{4}^{2}\\ & -0.0038_{5}^{2} \end{array}$$

(2) $$\begin{array}{ll} Y_{2}= & -4592.58+26.53X_{1}+5.68X_{2}+197.72X_{3}+767.33X_{4}+0.85X_{5}\\ & +0.0098X_{1}X_{2}-0.33X_{1}X_{3}+0.0015X_{1}X_{5}+1.09X_{2}X_{4}+3.43X_{3}X_{4}\\ & -0.04X_{3}X_{5}-1.56X_{4}X_{5}-0.04X_{1}^{2}-0.56X_{2}^{2}-11.68_{3}^{5}-571.34X_{4}^{2}\\ & -0.00083_{5}^{2} \end{array}$$

The F-test (*F*-value) and probability *p*-value test for the statistical significance of the coefficients in the regression equation indicated the interactions among the variables [27]. From the ANOVA results (Table S3), the *F*-values for models *Y*_1_ and *Y*_2_ were 49.42 and 32.82, respectively. The *p*-values were less than 0.0001, and the misfit terms *P* (*P*_1_ = 0.1720 and *P*_2_ = 0.0942) were greater than 0.05. The above results indicated that the model fit was very significant and the misfit term was insufficient to characterize the correlations between the adsorption conditions and the response values.

The coefficients of the linear terms (*X*_1_, *X*_4_, *X*_5_), interaction terms (*X*_3_*X*_5_, *X*_4_*X*_5_) and secondary terms ($X_{1}^{2}$, $X_{2}^{2}$, $X_{3}^{2}$, $X_{4}^{2}$, $X_{5}^{2}$) for *Y*_1_ and *Y*_2_ were significant (*p* < 0.05). This indicated that the adsorption temperature, adsorbent dosage, initial concentration of solution, interaction of pH with the initial concentration of solution, interaction of the adsorbent dosage with the initial concentration of solution, and the secondary terms of the factors were significantly correlated with the adsorption and removal of lead per unit of adsorbent. The coefficients of determination $R_{1}^{2}$ = 0.9677 and $R_{2}^{2}$ = 0.9677 indicated that the model was strongly correlated [28]. The $R_{1}^{2}$-Adj.$R_{1}^{2}$ = 0.0195 < 0.2, $R_{2}^{2}$-Adj.$R_{2}^{2}$ = 0.0029 < 0.2, and the coefficient of variation C.V._1_ = 4.940 < 10%, C.V._2_ = 5.540 < 10%, which indicated that the model was credible [29]. The signal-to-noise ratios of *Y*_1_ and *Y*_2_ were 28.34 and 28.21, respectively, which were greater than 4. This indicated that the above model was valid [30]. In summary, *Y*_1_ and *Y*_2_ provided better fits with the lead adsorption data of JK-BW and can be used for optimization of the JK-BW preparation process.

The ANOVA in Table S3 shows that the pH with the initial solution concentration and the adsorbent dosage with the initial solution concentration have a significant effect on adsorption capacity and adsorption rate, while the rest of the influences are not significant.

**Figure 2:**
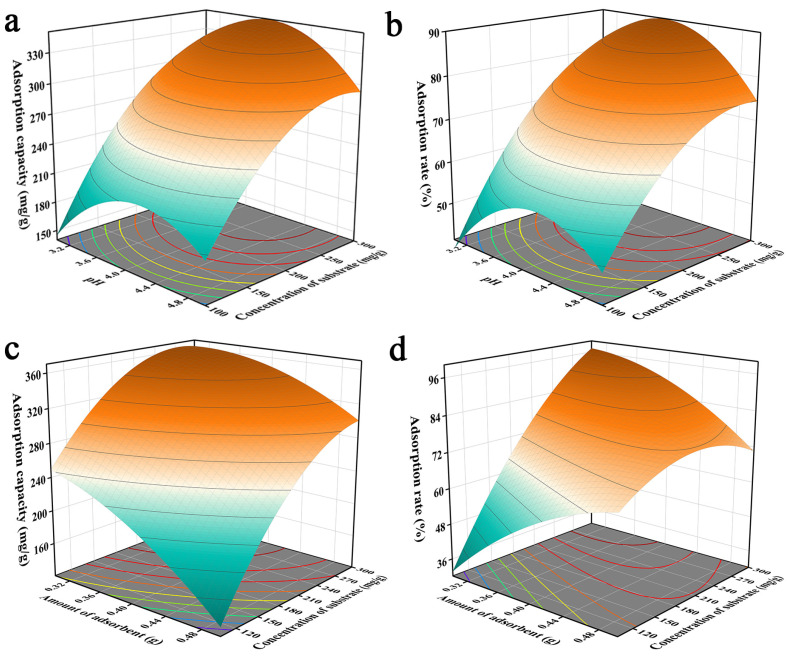
Effects of pH value and initial concentration of solution (**a**,**b**) and adsorbent dosage and initial concentration of solution (**c**,**d**) on the adsorption capacity of JK-BW.

The three-dimensional response surface plots provided a visual response indicating the effects of the optimized factors on the response values. The interaction of pH with the initial solution concentration is shown in Figure 2a,b. The unit adsorption of Pb^2+^ by JK-BW increased from 150.5 to 315.0 mg/g and the adsorption rate increased from 38.38% to 91.19% with increases in the initial concentration of the solution. The probability of Pb^2+^ collisions with JK-BW increased when the initial concentration of the solution was higher. It was inferred that the adsorption capacity of JK-BW was influenced greatly by the initial concentration of the solution. In addition, the unit amount and rate for adsorption of Pb^2+^ by JK-BW increased and then decreased with increases in the pH. This occurred because the high H^+^ content increased the competition with Pb^2+^ adsorption sites at the same solution initial concentration when the pH was low. This limited the binding of the material and Pb^2+^ with small unit adsorption and removal levels [28]. When the pH was increased to 4, more negatively charged ligands were exposed on the surface of the material; positively charged Pb^2+^ ions occupied more free binding sites, and adsorption was enhanced [31] with a rapid increase in the unit adsorption capacity and the removal rate. However, the unit adsorption capacity of the material decreased slightly at solution pH > 4. This is because the precipitation of insoluble metal hydroxides limits the true biosorption capacity [32,33]. In addition, the adsorbent dosage was also a fundamental factor affecting adsorption. As shown in Figure 2c,d, the more JK-BW was used, the less the unit adsorption amount decreased. This is occurred because overlapping active sites of the material reduced the adsorption area and increased the diffusion paths for lead [34]. However, the higher dosage of the adsorbent provided more adsorption sites, resulting in a significant increase in the removal rate. Similar findings were obtained by Parmar et al. [9].

The optimized adsorption conditions determined with the quadratic model and the response surface were an adsorption temperature of 306.5 K, an adsorption time of 7.82 h, pH = 4.05, an adsorbent dosage of 0.36 g/L, and an initial solution concentration of 296.8 mg/g. In order to facilitate practical operation and analyses, the adsorption conditions were adjusted to a temperature of 306 K, an adsorption time of 7.8 h, pH = 4, adsorbent dosage of 0.36 g/L, and an initial solution concentration of 300 mg/g. The theoretical adsorption capacity was 328.3 mg/g and the removal rate was 82.36% with these conditions. Pb^2+^ adsorption was carried out with these conditions to verify the predictive accuracy of the model. Finally, the optimized material was exhibited with a unit adsorption capacity of 325.3 mg/g (3.8-fold increase compared with the free cell) and an adsorption rate of 81.81%. The predictive accuracy was higher than 95%. As shown in Table S4, the weathered coal-immobilized microbial materials showed higher Pb^2+^ removal capacities than the nano-zero-valent iron-immobilized materials and biochar-immobilized materials prepared by Teng et al. [32], indicating that JK-BW is an effective Pb^2+^ adsorbent.

As shown in Figure S1, multiple adsorption-desorption experiments were carried out on JK-BW with 0.1 mol/L EDTA-Na_2_ used as the desorbent to investigate the regeneration stability of JK-BW on Pb^2+^. The unit adsorption capacity for Pb^2+^ on JK-BW was 325.6 mg/g and the removal rate was 84.94%, and it was clearly seen that the adsorption capacity decreased with increasing numbers of cycles. This may have been due to the loss of adsorption sites during adsorption/desorption or irreversible occupation of some adsorption sites [35]. After five cycles, the unit efficiency for the adsorption of Pb^2+^ by JK-BW remained stable at 269.8 mg/g with a removal rate of 70.96%. These results showed that JK-BW was readily regenerated properties and stabile.

### 2.3. Adsorption Kinetic Analysis

**Figure 3:**
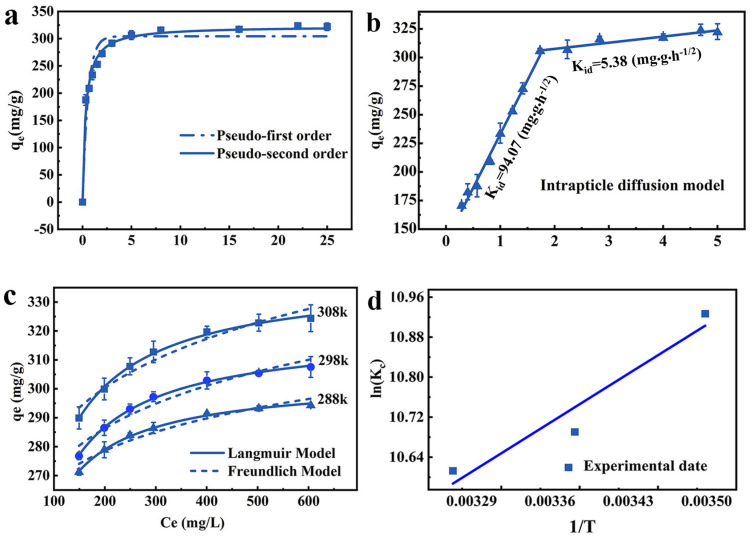
Adsorption kinetics analysis: pseudo-first-order kinetics model, pseudo-second-order kinetics model (**a**) and intraparticle diffusion model (**b**); thermodynamic analyses of isothermal adsorption: Langmuir and Freundlich models (**c**) and the effect of temperature on the equilibrium constant (**d**).

Pseudo-first-order and pseudo-second-order models are used to reveal the adsorption characteristics of JK-BW. The fitted models and parameters are shown in Figure 3a and Table 1. The R^2^ of the pseudo-second-order kinetic model is higher, indicating that it can better describe the Pb^2+^ adsorption performance of JK-BW. Therefore, the adsorption process is dominated by chemical action.

On this basis, the experimental results are fitted with the particle internal diffusion model to further explore the adsorption process of Pb^2+^. As is shown in Figure 3b, the Pb^2+^ adsorption amount of JK-BW and t^1/2^ can be fitted by two straight lines with different slopes over the whole time range. The fitting line does not pass through the origin, indicating that this process is not affected by a single diffusion factor [36]. The first stage lasts for the first 3 h, when approximately 291.7 mg/g (accounting for 90.59% of the saturated adsorption) of Pb^2+^ is adsorbed by JK-BW. The second stage occurs after 3 h when approximately 10% of Pb^2+^ is adsorbed on JK-BW and reaches adsorption equilibrium. The intraparticle diffusion parameters in Table 1 show that the slope constant behaviors are K_I_ > K_II_ for the two stages. According to this result, the adsorption of Pb^2+^ by JK-BW is divided into two stages: fast and slow. First, Pb^2+^ rapidly transfers from the solution to the surface layer of JK-BW and occupies many adsorption sites. The slow interaction is attributed to the internal diffusion of Pb^2+^ particles. Ding [37] and Zhao [36] obtained similar results by using biochar, porous organic materials, etc., to study the adsorption of Pb^2+^ in solution.

### 2.4. Isothermal Adsorption and Adsorption Thermodynamics

The impacts of solution concentration and temperature on Pb^2+^ adsorption are obtained for a fixed adsorbent amount of 0.36 g/L, initial solution pH of 4, and adsorption time of 24 h. According to the isothermal model, the experimental data were further fitted. Figure 3c and Table 2 show the fitting curves and parameters.

It should be noted that the coincidence degree of the Langmuir model to the experimental data under different temperature conditions is higher than that of the Freundlich model ($R_{1}^{2}$_adj_ > $R_{2}^{2}$_adj_), indicating that Pb^2+^ forms a uniform single-molecule adsorption layer on JK-BW [38]. The R_L_ values of JK-BW at different concentrations are between 0 and 1, indicating that JK-BW is favorable for Pb^2+^ adsorption under different temperature conditions. It is calculated from the Langmuir model that the adsorption amount of the material can reach 338 mg/g, which is higher than that of previously reported Pb^2+^ adsorption materials [36,39]. This result shows that the prepared JK-BW has excellent adsorption performance for Pb^2+^.

The thermodynamic research results and related parameters are shown in Figure 3d and Table 2. ∆G < 0 at different temperature conditions, indicating that the endothermic reaction can proceed spontaneously. |∆G| increases with increasing temperature, illustrating that the spontaneity of the reaction is positively correlated with temperature [36]. When ∆H > 0, it further increases the adsorption process, which is heat-absorbing. Values of 10 < ∆H < 30 indicate that Pb^2+^ adsorption on JK-BW may involve both physisorption and chemisorption [40]. This is consistent with the results of the analysis of the kinetics [29]. Values of ∆S > 0 indicate that the reaction proceeds in the direction of an entropy increase [30]; this phenomenon occurs because when the adsorption reaction occurs, Pb^2+^ transfers outwards from the bulk solution, resulting in an increase in entropy [41]. In summary, the adsorption of Pb^2+^ by JK-BW is a spontaneous reaction at high temperature, and the increase in temperature promotes the reaction.

### 2.5. Desorption Analysis

**Figure 4:**
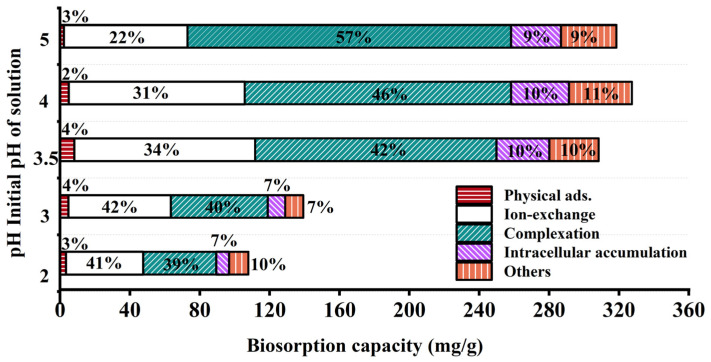
Relative contributions of different mechanisms.

After washing and drying JK-BW in adsorption equilibrium, the specimen is treated with DW, NH_4_NO_3_ and EDTA-Na_2_ and then fragmented. The relative contributions of the adsorption mechanisms at different pH values are shown in Figure 4. The release amount and desorption ratio of Pb^2+^ in DW are low (<5%), indicating that the Pb^2+^ adsorbed by JK-BW is not easily released and that the proportion of physical adsorption is very small. The desorption rate of Pb^2+^ by NH_4_NO_3_ is in the range of 22~42%. The release of Pb^2+^ via NH_4_NO_3_ is part of an ion-exchange mechanism, which is essentially the substitution of light metals, such as K^+^, Ca^2+^, Na^+^ and Mg^2+^, by Pb^2+^. In addition, the desorption of Pb^2+^ by EDTA-Na_2_ is above 39~57%. Pb^2+^ is strongly attached to JK-BW and not easily desorbed by NH_4_NO_3_, but it can be released by EDTA. This part of the adsorption is considered part of the complexation mechanism. The cumulative release of Pb^2+^ is 7~11% after the treatment of cell breakage, and this part of Pb^2+^ released by cell breakage is the intracellular cumulative part of biosorption. The rest is the precipitation mechanism. The adsorption of Pb^2+^ by JK-BW can be divided into five parts: physical adsorption, ion exchange, complexation, precipitation mechanism and microbial intracellular phagocytosis.

With the change in solution pH from 2 to 5, the unit adsorption amount of JK-BW for Pb^2+^ increases from 107.8 to 327.4 mg/g, which essentially occurs because the coordination of deprotonated functional groups with metal ions enhances the adsorption capacity of JK-BW [20,42]. Among the specimens, the amount of Pb^2+^ adsorbed by ion exchange increases from 44.14 to 103.6 mg/g, and the surface complexation increases from 41.80 to 185.2 mg/g. The results show that ion exchange and complexation have an important effect on biosorption [42]. According to the division of biological adsorption mechanisms by Srivastava et al. [43], the adsorption mechanism of Pb^2+^ by JK-BW is chemical adsorption; these findings are consistent with our previous research results on adsorption kinetics. Compared with the pre-immobilization period (Figure S2), the percentage of lead adsorption mechanism of JK-BW did not change significantly except for the additional intracellular accumulation fraction. However, its lead ion adsorption performance was improved by 74%. On the one hand, the immobilized strains and weathered coal carriers themselves have certain adsorption properties. More importantly, the vectors enhance the strain’s environmental adaptability, bioactivity and biodensity [18]. The abundant active functional groups on the surface of the strain (e.g., phosphate, carboxyl, carbonyl, amide groups, etc.) rapidly capture lead by physical adsorption, ion exchange, complexation, and intracellular phagocytosis [20,44], which greatly improves the lead adsorption performance of JK-BW.

### 2.6. Adsorption Mechanism

The adsorption mechanism of Pb^2+^ on JK-BW can be described by physical and chemical actions. On the one hand, BET and SEM analyses show that the complex microporous structure makes JK-BW easily contact Pb^2+^ ions, which is conducive to surface physical adsorption. On the other hand, kinetics, thermodynamics and desorption experiments show that the Pb^2+^ adsorption mechanisms of JK-BW are mainly chemical adsorption supplemented by physical adsorption [45]. To further analyze the adsorption mechanisms of JK-BW, EDS, FT-IR and XPS analyses are conducted before and after the adsorption of Pb^2+^.

**Figure 5:**
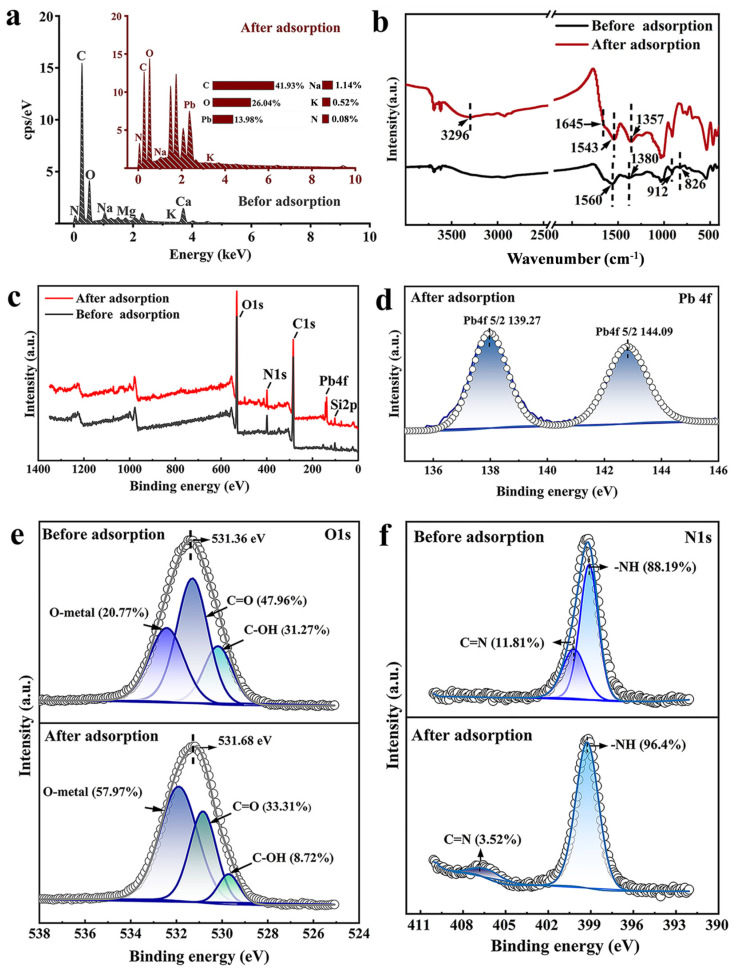
EDS spectra (**a**) and FT-IR spectra (**b**) of JK-BW before and after adsorption Pb^2+^; XPS survey of JK-BW before and after adsorption Pb^2+^ (**c**); Pb 4f spectra of JK-BW after adsorption Pb^2+^ (**d**); O 1s spectra (**e**) and N 1s spectra (**f**) of JK-BW before and after adsorption Pb^2+^.

The surface element distribution of JK-BW is shown in Figure 5a. The JK-BW surface contains rich elements, such as K, Ca, Na, Mg, C, N, and O, which are conducive to ion exchange and complexation reactions. After adsorption, a large amount of Pb^2+^ is enriched on the surface of JK-BW, which accounts for 14% of the surface element weight; this finding indicates that JK-BW is an effective material for adsorbing Pb^2+^. The FT-IR spectrum of JK-BW is shown in Figure 5b. Before the adsorption reaction, the peaks at approximately 1380 cm^−1^ indicate the existence of methyl structures –CH_3_ [46]. After the adsorption of Pb^2+^, a wide stretching vibration of −OH occurs at 3296 cm^−1^, while that at 1645 cm^−1^ appears as a weak stretching vibration of C=O [25]. At approximately 1357 cm^−1^, the half-peak width of –CH_3_ narrows, and the peak redshifts. It is suggested that hydroxyl groups, methyl structures, carbonyl groups and other active sites may affect the adsorption process of JK-BW [45]. The amide II coupling band is related to N–H and C–N with the absorption peak generally occurring between 1520 and 1570 cm^−1^. The peak at 1560 cm^−1^ is the bending of the N−H bond and the stretching of the C−N in the II band of the amide. After adsorption, this peak shifts to 1543 cm^−1^, which is presumably due to the reaction of Pb^2+^ with the amide group [47]. The β-ring of pyridine is considered a medium electron donor and corresponds to the peak at 826 cm^−1^, and it can bind Pb^2+^ through cation-π interactions [5]. FT-IR analysis shows that functional groups, such as hydroxyl, carboxyl, carbonyl, and amide groups, as well as the β-ring, are involved in the adsorption of Pb^2+^.

In addition, JK-BW before and after adsorption is analyzed by XPS. As shown in Figure 5c, there are only C 1s, N 1s, O 1s and Si 2p peaks observed before adsorption. The Pb 4f peak was added after adsorption, indicating that Pb^2+^ is successfully adsorbed on JK-BW. Additionally, curve fitting for the Pb 4f peak (as shown in Figure 5d) was evaluated according to Chen [48] and Wang [33]. PbCO_3_ (139.27 eV) and Pb–O (144.09 eV) are present, indicating that some precipitation is generated during adsorption. The O 1s spectrum of JK-BW is shown in Figure 5e. After adsorption, the O 1s binding energy of JK-BW is shifted from 531.36 to 531.68 eV, indicating that O^2−^ participates in adsorption. The O 1s spectrum of JK-BW can be divided into three peaks: C=O, C−OH and O–Metal. After adsorption, the relative amount of C−OH decreases from 31.27% to 8.72%, the C=O content decreases from 47.96% to 33.31%, and the O-Metal content increases from 20.77% to 57.97%. The results show that there is a chemical bond between Pb^2+^ and −COOH in JK-BW after adsorption. In addition, Figure 5f shows that the N1s binding energy of JK-BW after adsorption transfers from 399.12 to 400.19 eV, indicating that N participates in the adsorption of Pb^2+^. The N1s spectrum of JK-BW can be divided into two peaks, and its chemical forms are mainly −NH and C=N [1]. After adsorption, the relative amount of −NH continues to increase from 88.12% to 96.48%, and C=N exhibits a downward trend from 11.81% to 3.52% with a significant shift. This finding is caused by the change in the electron cloud density of the N atom during the formation of Pb^2+^ adsorption [49]. The possible adsorption mechanism is the formation of a coordination bond between Pb^2+^ and the amide group, which is consistent with the FT-IR analysis results.

**Figure 6:**
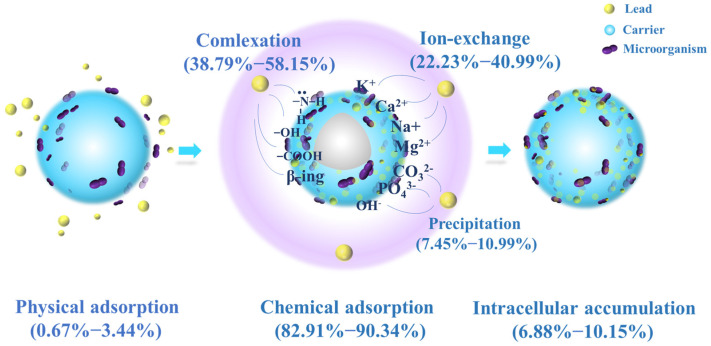
Hypothetical mechanistic diagram for the JK-BW and Pb^2+^ interaction.

In summary, the removal mechanism of Pb^2+^ includes physical adsorption, ion exchange, complexation, precipitation and intracellular accumulation, all of which may occur independently and cooperatively. The possible adsorption mechanism of JK-BW on Pb^2+^ is shown in Figure 6. (1) First, Pb^2+^ moves toward the solid–liquid interface and is fixed on JK-BW by a weak physical force. (2) The JK-BW surface provides many active groups, such as −OH, −COOH, CO−NH_2_ and β-rings. These groups rapidly adsorb Pb^2+^ through ion exchange and complexation. A small part of Pb^2+^ forms anionic coprecipitation with dissolved minerals. (3) The remaining Pb^2+^ accumulates in the cytoplasm after being engulfed by microorganisms.

## 3. Materials and Methods

The strain was screened from a heavy metal-contaminated site in Taiyuan City, Shanxi Province, and it was identified as Enterobacter Ludwig (CCTCCM2018095). The Pb^2+^ adsorption capacity and tolerance of the strain can reach 67 mg/g and 2000 mg/L, respectively.

JK was selected from the Yongxing coal mine in Jiao Kou County, Shanxi Province; it was ground into powder and sieved (<75 μm) to serve as the carrier material. The contents of heavy metals in the weathered coal were less than the corresponding risk screening values. The content of humic acid exceeded 30%, and the active functional groups were rich.

### 3.1. Adsorbent Preparation

Weathered coal was modified by ultrasonic crosslinking. First, 40 mL of deionized water (DW) was added to 5 g of dry weathered coal, the system pH was adjusted to 5. Then, 2.6 mL of ethylene amine was added, and the specimen was shaken through 40 kHz of ultrasonication for 65 min. Afterwards, the solid phase was repeatedly cleaned to make it neutral to the supernatant, and then the immobilized carrier JK-B was obtained after drying.

The adsorption immobilization method was used to synthesize JK-BW. The bacterial suspension cultured for 24 h (OD_600_ = 2.54) was injected into 10 mL/g carrier and immobilized at 25 °C for 18 h (thermostatic shaker, BSDYF2200, Boxun, Shanghai, China). Then, the mixture was centrifuged, and the sediment part of the lower layer was washed with normal saline to remove excess bacteria. The solid obtained by centrifugation was weathered coal-immobilized microbial material, which was labeled JK-BW [16].

### 3.2. Characterization

The micro-morphology and surface element composition of JK-BW were observed by scanning electron microscopy (SEM; MIRA4, Tesken, Brno-Kohoutovice, Czech Republic) and energy-dispersive spectroscopy (EDS, Xplore, Tesken, Czech Republic). The pore structure parameters of JK-BW were determined by a physical adsorption instrument through Brunauer-Emmett-Teller theory (BET; ASAP2460, Micromeritics, Norcross, GA, USA). Infrared spectroscopy (FT-IR; Nicolet iS20, Thermo Fisher, Waltham, MA, USA) and X-ray electron spectroscopy (XPS; K-Alpha, Thermo Fisher Economic, Waltham, MA, USA) were used to analyze the element contents and chemical bonds of JK-BW.

### 3.3. Batch Adsorption Experiments

A 1000 mg/L lead nitrate stock solution was configured with lead powder and nitric acid (refer to national standard HJ 491-2019 [50]). The solution was diluted to the desired concentration to simulate lead-filled wastewater.

A certain amount of JK-BW was weighed and placed in 50 mL lead nitrate solution, and the adsorption experiment was conducted on a constant temperature shaking table (28 ± 1 °C, 180 rpm/min). The effects of temperature (288~308 K), time (0~25 h), pH (3~5), amount of adsorbent (0.3~0.5 g/L) and initial concentration (100~600 mg/L) of solution on JK-BW adsorption performance were studied. We centrifuged the mixture after adsorption, and then the supernatant was taken and filtered for determination of Pb^2+^ content (AAS, 240/240FS, Agilent, Santa Clara, CA, USA). All experiments were set up in parallel three times with the unadded adsorbent used as the control to exclude the spontaneous precipitation and loss of Pb^2+^. The amounts and rates of Pb^2+^ adsorption by JK-BW under different conditions were calculated according to the literature [46].

### 3.4. Box-Behnken Design (BBD) and Response Surface Methodology

*Box-Behnken* design (BBD) was used to optimize the adsorption conditions of JK-BW. The response surface factors for temperature, time, pH, adsorbent dosage and initial substrate concentration were determined from the lead adsorption capacities of the materials in one-way pre-tests, respectively (Table S1).

### 3.5. Desorption Experiments

The mechanism of interaction between metal ions and adsorbents can be quantitatively evaluated by using appropriate desorbents [18,20]. Physically adsorbed Pb^2+^ is weakly bound and can be hydrolyzed. Pb^2+^ removed by ion exchange with light metals can be desorbed by NH_4_NO_3_, which is an ion exchange mechanism. Ions adsorbed by complexation mechanism tend to form complexes with functional groups and can be desorbed by EDTA-Na_2_. The amount accumulated intracellularly in microorganisms can be obtained by breaking the cells and measuring the amount of Pb^2+^ released. The remaining amount is attributed to precipitation and is calculated by subtracting the above quantities from the total adsorption amount one by one [18].

After adsorption equilibrium, the adsorbent was cleaned with deionized water many times to make it neutral. Then, the adsorbent was transferred to three desorption solutions—deionized water (pH = 4), NH_4_NO_3_ [51] and EDTA-Na_2_—and then shaken at 30 °C and 180 r∙min^−1^ for 4 h. Then, the adsorbent was placed on a vortex shaker for 10 min to break it up; thus, Pb^2+^ was fully released. The supernatant was collected for determination of Pb^2+^ concentration. The percentage desorption of Pb^2+^ was defined as follows [21]:

(3) $$Desorption\%=\frac{C_{e,elution}}{\left(C_{0}-C_{e}\right)}\times 100$$

where *C_e_*_,_*_elution_* (mg/L) is the content of Pb^2+^ in each desorption agent.

## 4. Conclusions

In this paper, weathered coal-immobilized microbial materials (JK-BW) for the efficient removal of lead from solution were obtained by immobilizing a lead-resistant functional strain on a weathered coal carrier. Box-Behnken response surface optimization experiments showed that the temperature, adsorbent dosage and initial concentration of the solution were significant factors affecting lead adsorption. The adsorption capacity of JK-BW was optimal when the temperature was 306 K, the adsorbent dosage was 0.36 g/L, and the initial concentration of the solution was 300 mg/g. The amount adsorbed reached 338.9 mg/g (308 K) mg/g, which was 3.8 times higher than that of the free cells, and the high adsorption capacity was maintained after four cycles of repeated use. The adsorption of Pb^2+^ by JK-BW was thermodynamically spontaneous and occurred via chemisorption-based monolayer adsorption. The adsorption mechanisms included ion exchange, complexation, precipitation, physical adsorption and microbial intracellular phagocytosis. Among these, the exchange interactions with K, Ca, Na, and Mg ions and complexation with the reactive functional groups –OH, –COOH, CO–NH_2_, and β-rings were the main mechanisms for Pb^2+^ adsorption on this material. In conclusion, the weathered coal immobilized microbial material showed a great potential for the removal of lead from the environment due to the good lead adsorption properties and renewability.

This study not only provides a material basis for the purification of lead-containing wastewater but also provides a direction for the high-value utilization of weathered coal. In addition, the adsorption, passivation and intracellular accumulation of JK-BW could jointly reduce the bioavailability of lead, which would provide the possibility of removing lead contamination from soil. Despite its potential, the environmental remediation of lead-contaminated water and soil through this material has not yet reached the stage of biotechnological application. The application of the material is the direction of our future research.

## Figures and Tables

**Table 1:** Adsorption kinetics and modeling parameters for particle diffusion of Pb^2+^ on JK-BW.

Sample	Pseudo-First-Order Model			Pseudo-Second-Order Model		
	K_1_ (h^−1^)	q_e_ (mg/g)	R^2^	K_2_ (h^−1^)	q_e_ (mg/g)	R^2^
JK-BW	1.816	303.97	0.939	9.628	326.4	0.999
Sample		Weber-Morris intraparticle diffusion model				
		K_3_ (mg/(g∙h^0.5^))	C ((mg/g)mg/g)		R^2^	
Ⅰ		94.07	139.01		0.99	
Ⅱ		5.38	296.78		0.91	

**Table 2:** Isothermal adsorption model constants and adsorption thermodynamic parameters.

Sample	T	Langmuir Model			Freundlich Model			R_L_	∆G (kJ/mol)	∆H (kJ/mol)	∆S (kJ/mol∙K)
	(K)	q_m_ (mg/g)	K_L_ (L/mg)	$R_{1}^{2}$	K_F_ (mg^(1 − n)^·L^n^/g)	n	$R_{2}^{2}$				
JK-BW	288	303.5	0.057	0.997	206.5	17.68	0.974	0 < R_L_ < 1	−26.164		
	298	319.7	0.043	0.998	195.1	13.81	0.975	0 < R_L_ < 1	−26.486	11.643	0.050
	308	338.9	0.040	0.997	197.3	12.61	0.987	0 < R_L_ < 1	−27.176		

## Data Availability

Data is contained within the article or Supplementary Material.

## Supplementary Materials

The following supporting information can be downloaded at https://www.mdpi.com/article/10.3390/molecules29030660/s1, Figure S1: Pb^2+^ adsorption capacities and removal rates of JK-BW for five regeneration cycles. Figure S2: Relative contributions of different mechanisms. Table S1: BBD factor level table, experimental design and results. Table S2: Pore structure parameters before and after immobilization. Table S3: Response surface analysis of variance (ANOVA) and credibility analysis of the regression equations. Table S4: Comparison of the Pb^2+^ adsorption activities of different microbial adsorbents. References [32,33,45,52,53] are cited in the Supplementary Materials.
