# Supplementary material for: Weathered Coal-Immobilized Microbial Materials as a Highly Efficient Adsorbent for the Removal of Lead
Source: Molecules. 2024 Jan 31;29(3):660. doi: 10.3390/molecules29030660 (PMC10856798; doi:10.3390/molecules29030660)
Supplement: Supplementary file 1 [file molecules-29-00660-s001.zip › molecules-2797088-supplementary.pdf]

Supporting Information

Weathered Coal-Immobilized Microbial Materials as a HighlyEfficient Adsorbent for the Removal of Lead

Zile Jiao [a,b], Chunhua Gao [a,b*], Jianhua Li [a,b], Jinjing Lu [a,b], Juan Wang [b], Lin Li [b], Xiaojing Chen [a,b,c*]

[a] Shanxi Agricultural University, Taiyuan 030031, Shanxi, PR China

[b] Shanxi Province Key Laboratory of Soil Environment and Nutrient Resources, Taiyuan 030031, PR China

[c] Institute of Loess Plateau, Shanxi University, Taiyuan 030006, China

[*] Corresponding authors.
E-mail addresses: chen-xiao-jing1985@sxau.edu.cn (X.J.Chen).

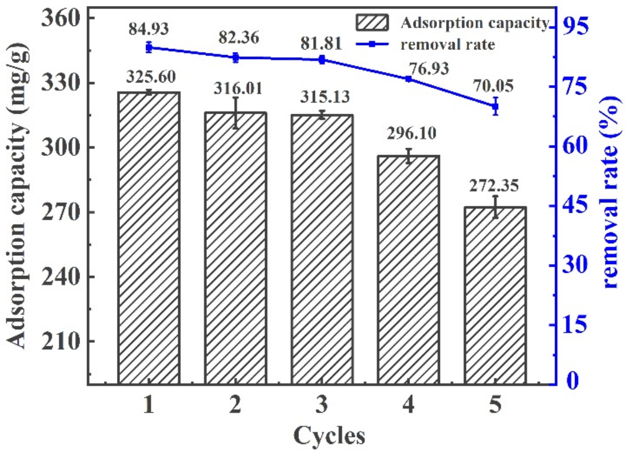

Figure. S1 Pb^{2+} adsorption capacities and removal rates of JK-BW for five regeneration cycles.

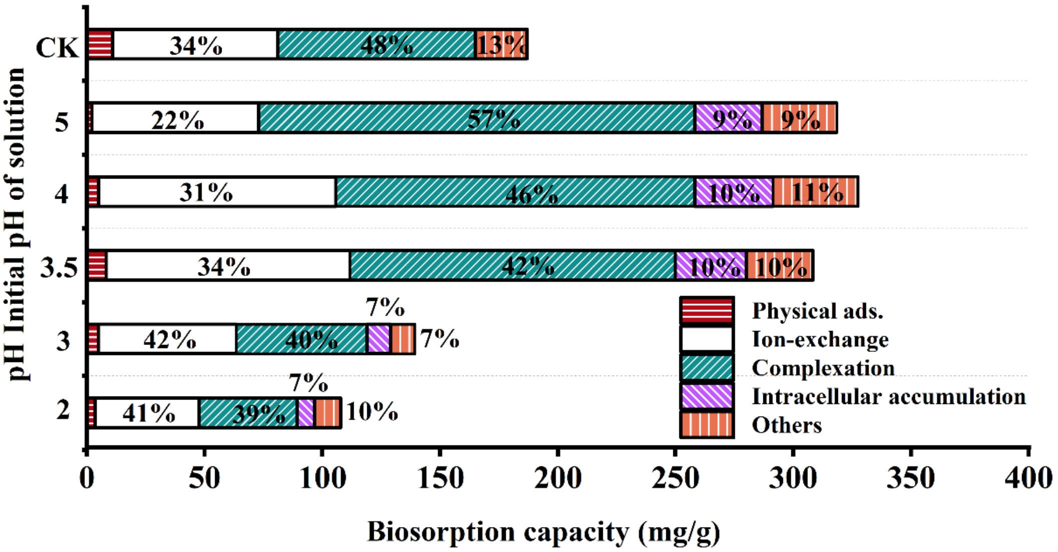

Note: CK indicates the relative contribution of each mechanism for the weathered coal carrier (JK-B) at pH = 4

Figure. S2 Relative contributions of different mechanisms.

Table S1. BBD factor level table, experimental design and results.

Variables	Units	Symbols	Levels		
			-1	0	1
Temperature	K	X_1	288	298	308
Time	h	X_2	4	8	12
pH	-	X_3	3	4	5
Amount of adsorbent	g	X_4	0.3	0.4	0.5
Concentration of substrate	mg/g	X_5	100	200	300

Run.	Coded levels of variables					Response value		Run.	Coded levels of variables					Response value	
	A	B	C	D	E	Y_1 (mg/g)	Y_2 (%)		A	B	C	D	E	Y_1 (mg/g)	Y_2 (%)
1	-1	-1	0	0	0	246.82	63.30	24	0	1	1	0	0	246.48	62.86
2	1	-1	0	0	0	268.79	68.63	25	-1	0	0	-1	0	321.88	82.09
3	-1	1	0	0	0	254.68	64.87	26	1	0	0	-1	0	344.11	87.76
4	1	1	0	0	0	281.79	71.77	27	-1	0	0	1	0	221.46	56.19
5	0	0	-1	-1	0	285.68	73.39	28	1	0	0	1	0	232.70	59.35
6	0	0	1	-1	0	304.46	77.45	29	0	0	-1	0	-1	150.48	38.38
7	0	0	-1	1	0	196.30	50.02	30	0	0	1	0	-1	184.60	47.39
8	0	0	1	1	0	218.99	56.52	31	0	0	-1	0	1	314.99	81.19
9	0	-1	0	0	-1	178.23	45.76	32	0	0	1	0	1	289.75	74.01
10	0	1	0	0	-1	179.14	45.45	33	-1	0	0	0	-1	187.40	47.29
11	0	-1	0	0	1	312.88	79.18	34	1	0	0	0	-1	193.70	48.99
12	0	1	0	0	1	312.88	79.91	35	-1	0	0	0	1	326.24	82.99
13	-1	0	-1	0	0	177.84	45.72	36	1	0	0	0	1	356.40	90.55
14	1	0	-1	0	0	305.92	78.22	37	0	-1	0	-1	0	325.72	83.40
15	-1	0	1	0	0	250.25	63.57	38	0	1	0	-1	0	328.32	83.42
16	1	0	1	0	0	251.98	64.03	39	0	-1	0	1	0	222.89	56.63
17	0	0	0	-1	-1	229.84	58.46	40	0	1	0	1	0	226.01	58.10
18	0	0	0	1	-1	136.05	34.84	41	0	0	0	0	0	307.34	79.01
19	0	0	0	-1	1	347.66	89.49	42	0	0	0	0	0	326.84	83.58
20	0	0	0	1	1	311.09	79.65	43	0	0	0	0	0	315.14	80.48
21	0	-1	-1	0	0	217.49	56.11	44	0	0	0	0	0	317.48	80.87
22	0	1	-1	0	0	218.27	55.90	45	0	0	0	0	0	323.13	82.30
23	0	-1	1	0	0	243.69	62.65	46	0	0	0	0	0	303.44	77.50

Note: X_1、X_2、X_3、X_4 and X_5 are the actual values of the temperature, adsorption time, pH, adsorbent dosage and initial concentration of solution, respectively.

Table S2. Pore structure parameters before and after immobilization

Sample	S_{BET}	Smic	Smes	V_t	V_{mic}	V_{mes}	D_{av}
	(m^2/g)	(m^2/g)	(m^2/g)	(cm^3/g)	(cm^3/g)	(cm^3/g)	(nm)
JK–B	1.736	1.890	1.232	0.0040	0.0007	0.0126	12.15
JK–BW	1.215	2.488	0.4540	0.0013	0.0009	0.0054	7.926

Table S3. Response surface analysis of variance (ANOVA) and credibility analysis of the regression equations

Sources of	ANOVA		Sources of	ANOVA	
	Y_1			Y_2	
	F-value	P-value		F-value	P-value
Model	49.42	< 0.0001	Model	32.82	< 0.0001
X_1	11.18	0.0024	X_1	8.62	0.0066
X_2	0.3529	0.5572	X_2	0.1982	0.6596
X_3	0.8859	0.3546	X_3	0.2772	0.6025
X_4	190.69	< 0.0001	X_4	5.35	0.0283
X_5	468.87	< 0.0001	X_5	345.24	< 0.0001
X_1X_2	0.0388	0.8453	X_1X_2	0.0442	0.8351
X_1X_3	4.01	0.0550	X_1X_3	3.06	0.0910
X_1X_4	0.1767	0.6774	X_1X_5	0.6193	0.4379
X_1X_5	0.8325	0.3693	X_2X_4	0.0547	0.8168
X_3X_4	0.0224	0.8822	X_3X_4	0.0339	0.8553
X_3X_5	5.15	0.0311	X_3X_5	4.73	0.0383
X_4X_5	4.79	0.0372	X_4X_5	69.92	< 0.0001
X_1^2	9.87	0.0039	X_1^2	11.38	0.0022
X_2^2	56.38	< 0.0001	X_2^2	50.16	< 0.0001
X_3^2	100.22	< 0.0001	X_3^2	86.02	< 0.0001
X_4^2	15.59	0.0005	X_4^2	20.57	< 0.0001
X_5^2	72.27	< 0.0001	X_5^2	42.92	< 0.0001
Lack of it	2.37	0.1720	Lack of it	3.30	0.0942

Credibility analyses of the regression equations

Index	Std.dev	Mean	C.V.%	R^2	Adj. R^2	Pre. R^2	Ade Pre
Y_1	13.07	264.59	4.94	0.9677	0.9482	0.8976	28.3450
Y_2	3.72	67.12	5.54	0.9522	0.9232	0.8354	28.2190

Note: Y_1 represents the lead adsorption capacity of the restorative, Y_2 represents the lead removal rate of the restorative.

Table S4. Comparison of the Pb^{2+} adsorption activities of different microbial adsorbents

Absorbent	Adsorption capacity (mg/g)	Removal rate (%)	References
Bacteria beads loaded with nanoscale zero valent iron	27.50	93	[1]
Immobilized cells of biochar	89.39	-	[2]
Efficient adsorption of lead microbial cells	138.88	57.21	[3]
Phosphate-embedded calcium alginate beads	236.16	94.2	[4]
Spongy microbe-based biosorbents	345.02	61.54	[5]
Weathered Coal-Immobilized Microbial Materials	325.32	81.81	This study

References:

[1] Teng Z, Shao W, Zhang K, *et al*. Enhanced passivation of lead with immobilized phosphate solubilizing bacteria beads loaded with biochar/ nanoscale zero valent iron composite[J]. J Hazard Mater, 2020,**384**:121505, https://doi.org/10.1016/j.jhazmat.2019.121505

[2] Vaghela D R, Pawar A, Panwar N L, et al. Modelling and Optimization of Biochar-Based Adsorbent Derived from Wheat Straw Using Response Surface Methodology on Adsorption of Pb^{2+}[J]. Int J Environ Res, 2023,17(1), https://doi.org/10.1007/s41742-022-00498-3

[3] Samimi M, Shahriari-Moghadam M. Isolation and identification of Delftia

lacustris Strain-MS3 as a novel and efficient adsorbent for lead biosorption: Kinetics and thermodynamic studies, optimization of operating variables[J]. Biochem Eng J, 2021,**173**:108091, https://doi.org/10.1016/j.bej.2021.108091

[4] WANG Y, YAO W, WANG Q, *et al*. Synthesis of phosphate-embedded calcium alginate beads for Pb(II) and Cd(II) sorption and immobilization in aqueous solutions[J]. T Nonferr Metal Soc, 2016,**26**(8):2230-2237, https://doi.org/10.1016/S1003-6326(16)64340-6

[5] Wang N, Qiu Y, Xiao T, *et al*. Comparative studies on Pb(II) biosorption with three spongy microbe-based biosorbents: High performance, selectivity and application[J]. J Hazard Mater, 2019,**373**:39-49, https://doi.org/10.1016/j.jhazmat.2019.03.056
